# Prevalence and factors associated with burnout among junior medical doctors at a South African tertiary public sector hospital

**DOI:** 10.11604/pamj.2024.47.208.41865

**Published:** 2024-04-24

**Authors:** Malebo Kgatle, Jerry George, Fiona Dominic, Pieter De Jager

**Affiliations:** 1 Department of Pediatrics, Paarl Regional Hospital, Western Cape, South Africa,; 2 Department of Neurosciences, School of Clinical Medicine, University of Witwatersrand, Johannesburg, South Africa,; 3 Department of Anatomical Pathology, University of Witwatersrand, Johannesburg, South Africa,; 4 Department of Anesthesia, University of Witwatersrand, Johannesburg, South Africa

**Keywords:** Burnout, medical interns, emotional exhaustion, depersonalization, personal achievement

## Abstract

**Introduction:**

burnout is a syndrome characterized by emotional exhaustion, depersonalization and emotional exhaustion that occurs due to exposure to stressful conditions over a long period. It can lead to poor job performance, apathy, and lack of productivity. This study looks at the prevalence of burnout in medical interns in a tertiary hospital in South Africa and the factors that may contribute to burnout.

**Methods:**

an analytical cross-sectional study was conducted. Medical interns working in Chris Hani Baragwanath Hospital in 2019 were invited to participate. The participants filled questionnaire that had demographic information, the Maslach Burnout Inventory Scale, a scale to rate the rotations that they believed contributed towards their burnout and factors they think contributed towards their burnout. Our data was analyzed using Stata.

**Results:**

out of a possible 165 potential participants, 101 medical interns enrolled. 95% of the participants reported burnout. Statistically significant factors contributing towards burnout were lack of resources and poor relations with support staff and senior staff. The medical rotation that was reported by the participants to contribute most towards their burnout was internal medicine.

**Conclusion:**

burnout in this population of medical interns is alarmingly high. Higher than reported in similar studies in South Africa and internationally.

## Introduction

Burnout emerged as a concept in the 1970s and it was used to explain people's negative experiences with work [1]. Burnout is a syndrome defined by: emotional exhaustion, depersonalization and reduced personal accomplishment that occur as a result of an extreme and long-term response to stressful situations [2,3]. Features of emotional exhaustion include fatigue, insomnia, and impaired concentration, loss of appetite, anxiety and depression, features of depersonalization: loss of enjoyment, pessimism, detachment and isolation, features of reduced personal accomplishment: apathy, increased irritability and lack of productivity [4]. In recent years burnout has come under the spotlight as a social problem worthy of attention and addressing. In the medical doctor population, it has been shown to be associated with increased rates of depression, anxiety, drug and alcohol abuse and suboptimal patient care [5-7]. Studies have been done locally and internationally on the prevalence of burnout. These include a study done by Rossouw *et al*. which showed there was at least 76% [8]. Another study done in Bloemfontein showed at least a 47% prevalence of burnout, this study was done across 4 levels of hospital care and included medical officers and registrars with varying years of experience in the medical field [9].

Internationally a similar study was done in India. This study recruited medical interns and residents and showed that surgical residents had the highest prevalence of burnout (57.9%) and that medical interns had a 55.2% prevalence of burnout [5]. An Irish study was done in 2 academic hospitals but only assessed 22 interns. The intern population was found to have a moderate level of burnout [10]. There are several scales used to measure burnout the Copenhagen Burnout inventory, Oldenburg Burnout inventory and Maslach Burnout inventory. However, the Maslach is the most commonly used scale and explores the three dimensions comprehensively and is considered the gold standard [11].

There has been little attention given to the prevalence of burnout among medical interns working in South Africa. Chris Hani Baragwanath Academic Hospital (CHBAH) is the third largest hospital in the world and the largest in the Southern Hemisphere with a bed capacity of over 3000 beds. The cohort of interns working at this particular hospital have a massive burden to deal with. They are required by the Health Professionals Council of South Africa (HPCSA) to rotate through various disciplines for approximately 4 months. While rotating through these teaching blocks they are required to work overtime in addition to their normal working hours

**Aims and objectives:** to determine the prevalence of burnout among junior medical doctors (i.e., first and second-year medical interns) working at Chris Hani Baragwanath Academic Hospital; to identify factors associated with burnout among junior medical doctors at Chris Hani Baragwanath Academic Hospital and provide the results of the study to the psychology and psychiatry departments of Chris Hani Baragwanath Hospital to attempt sessions for coping strategies in this population

## Methods

**Study design:** an analytical cross-sectional study was conducted.

**Study setting:** this study took place at Chris Hani Baragwanath Academic Hospital (CHBAH).

**Participants:** all medical interns employed at CHBAH employed between 2018 and 2019 were invited to participate. All medical interns were to be older than 18 and registered with the Health Professional Council of South Africa. A total of 165 potential participants were invited to participate.

**Data source:** a questionnaire was developed which contained the Maslach Burnout Inventory (MBI) scale as well data on demographics, which rotations the interns had done, their motivation for becoming medical doctors and which factors the medical interns felt contributed towards their burnout. The Maslach Burnout Inventory (MBI)Scale (Human Services Scale) was used to assess for burnout. The MBI is considered the scale of choice when researching burnout [12]. The MBI measures 3 areas of burnout, emotional exhaustion (EE), depersonalization (DP) and personal achievement (PA) and it can grade the level of burnout in each section into “low”, “moderate” and “high”. The scale consists of 22 questions each rated from 0-6. High EE is points >26, high DP is >13, high DP is <31 points. A person is considered clinically' burnt-out if they score ‘high’ levels in any of the EE, DP and PA.

**Study size:** based on the available literature we expect a burnout prevalence of 65% among a study population of 165 interns. At a confidence interval of 90% and a power of 5%, we would require a sample size of 100.

**Statistical methods:** data was collected via a self-administered questionnaire which included demographics, MBI, which rotations they had completed at the time of enrollment, which rotations they believed contributed most to their burnout, and factors they believe contributed towards their burnout. The data was captured in Microsoft Excel. The data was then exported into a Stata® [Stata Corporation, College Station United States of America] for analysis. The residents who completed the burnout questionnaire were described in terms of demographic and training-associated variables. Categorical variables were described as frequencies and percentages. Continuous variables were described as medians with interquartile ranges. Appropriate graphs and charts were used to visualize the data. Burn-out was defined as scoring high/moderate on all three burn-out domains - depersonalization, emotional exhaustion and personal achievement. Frequencies and percentages were used to describe the occurrence of burn-out among the residents overall, by the different burn-out area/domain, by age, sex and training or work-related experiences. Chi-squared tests were used to determine the association between demographic and training/work-related experiences and burnout. A p-value <0.05 represented a statistically significant association between the variables and having burn-out

**Inclusion criteria:** the study included first- and second-year interns registered with the HPCSA; and employed at Chris Hani Baragwanath Academic Hospital; and older than 18 years; and who has provided signed informed consent.

**Potential bias:** two of the researchers were medical interns in Chris Hani Baragwanath Academic Hospital.

**Ethical considerations:** the study was approved by the Human Resources and Ethics Committee (HREC) of the University of Witwatersrand as well as the CEO of CHBAH. Clearance certificate number M181179. The anonymity of the participants was maintained. No identifying data was collected. Participation was completely voluntary and the participants were allowed to withdraw at any point without any adverse event if they chose to withdraw participation.

## Results

**Participants:** we interviewed 101 participants in Chris Hani Baragwanath Hospital out of a possible 165 (Response rate of 61%). Table 1 shows the demographic profile. There were 63 females (63%) and 38 males (37%). 17 participants were married)17%), 1 participant was divorced and 83(82%) were single. 48 (47,5%) participants had done their undergraduate medical degree from the University of Witwatersrand (they had prior experience at CHBAH) and 53 participants had studied in other universities (no prior experience at CHBAH).

**Table 1:** demographic details of participants

Gender	N	%
Male	38	37%
Female	63	63%
**Marital status**		
Single	84	84%
Married	17	16%
**University studied**		
Wits	48	47%
Other	53	53%
**Residence**		
Gauteng	38	37%
Other	63	63%

**Outcome data:** the overall prevalence of burnout in our cohort was 95%. Based on the MBI score, participants were categorized into high, moderate and low burnout in the 3 categories (Emotional exhaustion, depersonalization and personal achievement) (Table 2).

**Table 2:** percentage of burnout in different aspects of MBI

	Emotional Exhaustion	Depersonalization	Personal Achievement
High Burnout	79%	45%	95%
Moderate Burnout	17%	43%	5%
Low Burnout	4%	12%	0%

**Figure 1:**
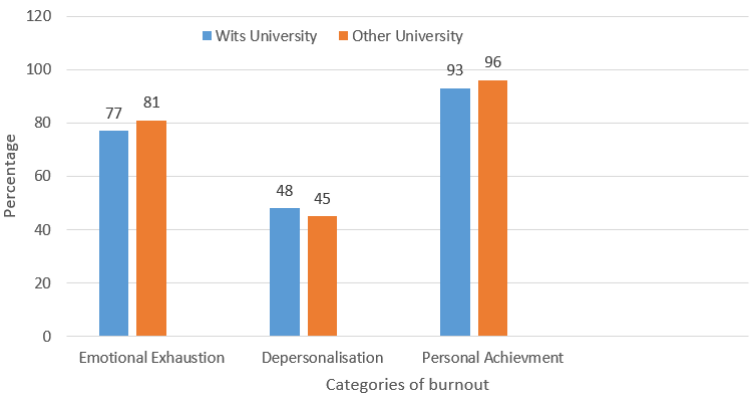
pie chart comparing the different aspects of burnout in Wits University graduates vs other university graduate

**Descriptive data:** regarding emotional exhaustion 79% of participants experienced high burnout, 17% experienced moderate burnout and only 4% experienced low burnout. On depersonalization, 45% of participants experienced high burnout, 43% experienced moderate burnout and 12% low burnout. Personal accomplishment had 95% of participants experiencing high burnout and 5% experiencing moderate burnout. 67 participants were doing their second year of internship and 34 were still doing their first year of internship. In CHBAH there are set rotations for first and second years. First years rotate through internal medicine, Obstetrics and Gynecology Surgery and Pediatrics then in their second year they anesthesia, family medicine. Regarding age, 62 participants were aged between 20 - 25 and 39 participants were between ages 26-30. Ninety-two percent (92%) of the participants aged between 20-25 were burnt-out vs 100% of the participants 25-30 (P: 0,06) Male vs female percentage burnout was 95% vs 95% (P 0,9). Regarding the year of internship 88% of first years reported burnout vs 98% of second years (P: 0.025) (Figure 1).

**Factors contributing to their burnout:** we looked at Long working hours, large patient load, lack of resources, lack of support from seniors, lack of resources, going beyond their job description. Participants scaled this from 1 - 5 (1 being least important and 5 being more contributory to burnout). For long working hours, 62 participants reported 5/5 and 30 participants 4/5 (P: 0.8). Regarding patient load 61 participants scored it 5/5 and 20 participants 4/5 (P: 0.77). Insufficient support from seniors, 30 participants scored it 5/5. Poor working relationship with other staff support 27 participants scored it 5/5 (P: 0.002). Lack of resources, 53 participants rated it 5/5(P 0.07). Going beyond the job description 49 participants rated it 5/5(0.6). Insufficient time spent with family and friends, 30 participants rated it 5/5 (P: 0.014). Regarding the medical rotation contributing the most towards burnout. We had asked the participants to rate it from 1 to 8. 1 being most contributing and 8 being least contributory.

As regards to internal medicine, 84 participants had completed internal medicine. 48/84 participants considered it the most contributory towards burnout. Of the 48 participants who completed internal medicine, 2 participants did not have burnout and 46 participants had burnout(P: 0.9).

Regarding surgery, 88 participants had completed surgery rotation. 18/88 considered surgery the most contributory towards burnout and 39/88 considered it seconds most important (P: 0.97). Obstetrics and Gynecology, 82 participants had completed the rotation. 11/82 considered it most contributory towards burnout and 11/82 considered it seconds most contributory (P: 0.75). Pediatrics, 31 had completed it. 0/30 considered it most contributory. 8/30 considered it 4^th^ most contributory. Orthopedics, 43 completed the rotation. 2/48 considered it most contributory. In family medicine, 54 participants had completed the rotation and 2/54 considered it most contributory to burnout (P: 0.3) Anesthesia 42 participants completed it and 0/42 reported it most contributory to burnout. Psychiatry, 36 participants had completed the rotation and 0/36 reported it most contributory to burnout.

**Figure 2:**
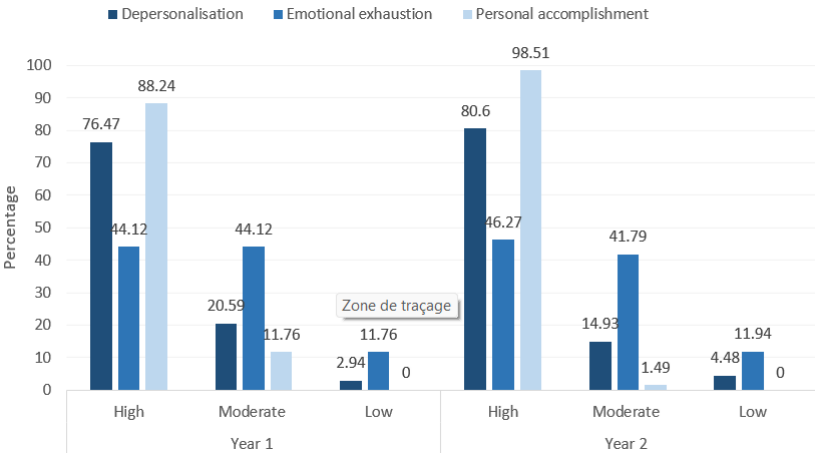
pie graph comparing the percentage of burnout in first and second year interns on the various categories of burnout

Regarding the university in which the participants studied for their medical degree,48 participants (47%) had studied in Wits University and 53(53%) participants had studied in another University. (Figure 2). Wit's participants, 44 participants (91%) burnout. Participants who studied elsewhere, 52 of them experienced burnout (98%), P value 0.76.

## Discussion

We interviewed 101 participants of the total 165 medical interns. High scores of burnout were recorded in 79% of participants based on emotional exhaustion, 95% based on personal achievement and 48% on depersonalization scores. Based on the MBI scale, a person Is said to have clinically indicative burnout if he/she scores high scores in emotional exhaustion or depersonalization [2]. This means 95% of medical interns in CHBAH experience burnout (Table 2). This is markedly higher than the 76% reported by the study In the Western Cape as well as much higher than the Bloemfontein study which reported 47% burnout [8,9]. We also report a higher percentage of burnout than international studies done in India and Ireland [5,10]. We need to also keep in mind that our study only looked into medical interns. Medical interns are the most junior doctors.

Looking at the demographics in our study (Table 1), we see that there is slightly more burnout reported in the age group of 20-25 vs the 25 - 30 age group. As well as higher burnout reported in the second-year participants as compared to the first. These second-year participants had all already completed Internal Medicine, Surgery and Obstetrics and Gynecology and these from the reporting done by participants were the rotations most contributing towards their burnout. When looking at the factors associated with burnout, the statistically relevant factors were the relationship with seniors and other staff as well as lack of resources. But of note majority of participants reported that the factor most contributory towards their burnout were large patient load, long working hours and going beyond their job. Regarding the rotation that the participants contributed most towards their burnout, Internal medicine was the most reported, followed by surgery then Obstetrics and Gynecology.

The level of burnout in the medical interns at CHBAH is alarmingly high. Overall, 95% of medical interns who participated in our study reported burnout. The rotation that was reported to be most associated was internal medicine followed by surgery. These rotations have been known to have very long hours and the patient load is very high thus resulting in burnout. This is alarmingly high and this needs to be addressed. We recommend hiring of more medical interns, regulated working hours, debriefing sessions for the medical interns

**Limitations:** there were some participants on leave and some did not want to participate.

## Conclusion

Out of the 101 participants, 95% of the population experienced burnout. This is much higher than what is described in other local studies and international studies. This study only included medical interns based at Chris Hani Baragwanath Hospital. This level of burnout is worrying and these medical interns should be offered supportive measures to support them during the years of internship. Supportive measures could include regular debriefing sessions with colleagues and seniors, better relationships with seniors (create mentorship programs) or psychologist sessions.

### What is known about this topic

- *Burnout is a problem that causes decreased productivity;*
- *Burnout is present in medical doctors as has been reported in international and local studies (Bloemfontein and Cape Town)*.

### What this study adds

- *The prevalence of burnout in this medical intern population is higher than reported in other studies, nationally and internationally;*
- *The medical rotation reported to contribute most towards this cohort's burnout was internal medicine;*
- *The statistically significant factors associated with burnout were lack of resources and relationship with senior and other staff*.
